# Evaluation of Bleb Fluid After Baerveldt Glaucoma Implantation Using Magnetic Resonance Imaging

**DOI:** 10.1038/s41598-017-11054-x

**Published:** 2017-09-12

**Authors:** Kentaro Iwasaki, Masayuki Kanamoto, Yuji Takihara, Shogo Arimura, Yoshihiro Takamura, Hirohiko Kimura, Masaru Inatani

**Affiliations:** 1 Department of Ophthalmology, Faculty of Medical Sciences, University of Fukui, Fukui, Japan; 2 Radiological Center, University of Fukui Hospital, Fukui, Japan; 3 Department of Radiology, Faculty of Medical Sciences, University of Fukui, Fukui, Japan

## Abstract

We evaluated bleb fluid images taken after Baerveldt glaucoma implantation. T2-weighted images of bleb fluid were scanned with 3 Tesla magnetic resonance imaging in 52 patients who had undergone tube-shunt surgery using the 350-mm^2^ endplate Baerveldt glaucoma implant; three-dimensional images were constructed from these images. Bleb fluid images were classified into either a layer of bleb fluid on either side of the endplate (double bleb layer group; n = 24) or one layer outside the endplate (single bleb layer group; n = 28). Despite there being no correlation between the bleb volume and the postoperative IOP (*r* = −0.080; *P* = 0.57), the double bleb layer group had significantly lower postoperative IOPs than the single bleb layer group (12.3 ± 3.8 mmHg vs. 14.7 ± 4.1 mmHg, respectively; *P* = 0.033). The single bleb layer was significantly related to higher numbers of prior intraocular surgeries (relative risk = 2.85; *P* = 0.0014). Formation of a layer of bleb fluid on either side of the endplate may have resulted in the lower postoperative IOPs after Baerveldt glaucoma implantation. Repeated intraocular surgery adversely affects formation of the double bleb layer.

## Introduction

Tube-shunt surgery using glaucoma drainage implants has become increasingly popular in refractory glaucoma in patients who have undergone one or more intraocular surgeries^1^. The Tube Versus Trabeculectomy Study showed that tube-shunt surgery using the Baerveldt glaucoma implant with a 350-mm^2^ endplate offers a higher success rate in patients that have undergone prior trabeculectomy and/or cataract surgery than those who underwent trabeculectomy alone^2^. Because the intraocular pressure (IOP)-lowering effect of trabeculectomy depends on the formation of a filtering bleb in the conjunctiva near the corneal limbus, surgical success is greatly affected by any prior surgical incisions in the conjunctiva^3^. Furthermore, because the Baerveldt glaucoma implant has a tube that is inserted into the eye and an endplate placed in the posterior region of the eye, the filtering bleb is formed around the endplate^4^. The surgical outcome of this procedure appears to be less affected by conjunctival surgical incisions than trabeculectomies, because the bleb forms in the posterior region of the eye compared to the bleb formation after trabeculectomy.

Unlike the filtering bleb that results from trabeculectomies, the bleb fluid structure created by Baerveldt glaucoma implantation cannot be assessed by the instruments used in standard ophthalmic examinations such as anterior-segment optical coherence tomography or ultrasound biomicroscopy because of its posterior location. Magnetic resonance imaging (MRI) is a useful tool for noninvasively evaluating bleb fluid structure after tube-shunt surgery^5–8^. T2-weighted images can be used to visualize and quantify the bleb fluid around the endplate, and the bleb image is shown as a dark circumlunar band (endplate) surrounded by a pocket of water density (bleb fluid)^7^. A recent cross-sectional study using 3 Tesla MRI showed that the bleb structure consists of two fluid layers divided by the endplate^8^. These previous case series have demonstrated that filtering bleb volume is negatively correlated with postoperative IOP^7, 8^. However, one study included eight cases with Ahmed glaucoma valve and Molteno implants^7^, whereas another study included 27 cases with Ahmed glaucoma valve and Baerveldt glaucoma implant with a 350-mm^2^ or 250-mm^2^ endplate^8^. IOP-lowering effect of these implants is affected by the size^9–11^, material^12^, and internal valve of the endplate^10, 11^. Although a larger endplate appears to be associated with a larger bleb volume, comparisons between the 350-mm^2^ and 500-mm^2^ endplate Baerveldt glaucoma implants and between the 350-mm^2^ and 250-mm^2^ endplate Baerveldt glaucoma implants showed a higher success rate for the 350-mm^2^ endplate than that for the 500-mm^2^ endplate^13^ and a comparable success rate between the 350-mm^2^ and 250-mm^2^ endplates^14^. Moreover, 11 eyes with the Ahmed glaucoma valve showed no correlation between the filtering bleb volume and postoperative IOP^15^. To assess these controversies, it is necessary to visualize bleb images and quantify bleb volumes in a large sample population implanted with the same glaucoma drainage implants having same size endplates using MRI.

The purpose of the present study was to assess the bleb structure after tube-shunt surgery using a 350-mm^2^ endplate Baerveldt glaucoma implant, and to determine the parameters of bleb images that correlated with postoperative IOP in a large sample size.

## Methods

### Patient Selection

This cross-sectional case series was approved by the institutional review board of Fukui University Hospital, Fukui, Japan. The protocol followed the guidelines of the Declaration of Helsinki. Written informed consent was obtained from all patients after detailed explanation of the procedures involved.

Patients previously treated with Baerveldt glaucoma implantation at the Fukui University Hospital were recruited. Orbital MRIs were obtained more than 6 months after surgery between December 1, 2014 and March 31, 2016. Patients aged 20 years or older who underwent tube-shunt surgery with the Baerveldt glaucoma implant BG101-350 or BG102-350 (Abbott Medical Optics, Abbott Park, IL) were included. Patients treated with other endplate types of the Baerveldt glaucoma implant (BG103-250); those who underwent Baerveldt glaucoma implantation within 6 months or less; those who had undergone other tube-shunt surgeries; patients with contraindications for MRI including patient intolerance, cardiac pacemakers, or presence of tattoos or metal in their bodies; or those who showed postoperative complications such as a bleb leak or hypotony at the time of MRI were excluded. The three-dimensional images were constructed using a volume-rendering tool software (Advantage Workstation VolumeShare7 Ext5; GE Healthcare, Milwaukee, WI).

### Surgical Procedures

One surgeon (MI) performed all surgeries, and the surgical procedure was performed as described in this section. In brief, all tube-shunt surgeries were performed using a 350-mm^2^ Baerveldt glaucoma implant (BG101-350 or BG102-350). The silicone tube was completely occluded with a 8-0 absorbable vicryl (Coated VICRYL; Ethicon, Somerville, OH) suture to minimize the risk of early postoperative hypotony. A fornix-based conjunctival flap was created after the administration of subconjunctival and sub-Tenon’s xylocaine anesthesia. The silicone plate was preferentially placed in the superotemporal scleral quadrant. If the superotemporal quadrant had intensive surgical conjunctival scarring, the plate was placed in one of the other quadrants. After the endplate was placed under the rectus muscles, it was fixed on the scleral surface with a 7-0 nylon about 10 mm from the corneal limbus. A scleral tunnel was created into the anterior chamber with a 23-gauge needle to insert the tube into the anterior chamber. As for tube insertion into the vitreous cavity, a 20-gauge needle was used to penetrate the vitreous space. Then, the tube with the Hoffmann elbow (BG102-350) was trans-sclerally inserted into the vitreous space and sutured to the sclera with a 9-0 nylon. To reduce the frequency of early postoperative IOP elevation, Sherwood slits were created in the tube with the needle of the 9-0 nylon. The tube was covered with a patch graft of preserved sclera supplied by the eye bank. The scleral patch and the conjunctival flap were sutured using a 9-0 nylon and 8-0 absorbable vicryl suture, respectively.

All patients received similar postoperative topical medication, with 0.5% levofloxacin for 3 weeks and 0.1% betamethasone sodium phosphate for 6 months.

### Data Collection

Patient data including sex, age, type of glaucoma, axial length, preoperative IOP, the endplate position, postoperative IOP, history of previous intraocular surgery, and the number of glaucoma medications were collected.

### Magnetic Resonance Imaging

All scans were obtained more than 6 months after surgery. High-resolution orbital images were obtained using a 3 Tesla scanner (Discovery MR 750 3.0 T; GE Healthcare, Milwaukee, WI) in combination with a 32-element phased-array head coil with fast imaging sequences employing steady-state acquisition cycled phases (FIESTA-C). The imaging parameters were as follows: repetition time, 5.6 ms; echo time, 2.7 ms; field-of-view, 180 mm; matrix size, 320 × 288; and a slice thickness of 0.6 mm with an overlap thickness of 0.3 mm, resulting in an in-plane resolution of 0.56 mm × 0.63 mm^8^. Images of the coronal plane were obtained perpendicular to the plane containing both optic nerves. Acquisition time for the whole scan was approximately 3 min. Subjects were repeatedly asked to avoid unnecessary movements during scanning. Baerveldt glaucoma implants are not ferromagnetic and are therefore MRI safe. The specific absorption rate was calculated to be less than 2.0 W/kg, which is classified as “less invasive” for patients. None of the patients felt warmth from the implants during the scans.

### Measurement of Bleb Volume

MRI scans obtained were analyzed using the ImageJ software (available at http://rsb.info.nih.gov/ij/; developed by Wayne Rasband, MD, PhD, National Institutes of Health, Bethesda, MD) on a personal computer^8^. Image data were saved as DICOM files and imported into the ImageJ software. A threshold was applied to identify individual blebs in each slice, and bleb area was calculated using the ImageJ software by two independent observers (KI, SA). For an automatic threshold selection, Otsu thresholding^16^ was chosen. Interobserver reliability test was obtained by an intraclass correlation coefficient (ICC) from a 2-way mixed effect model. The ICC was 0.984 (95% confidence interval, 0.973 – 0.991). The mean values of bleb areas quantified by two observers were integrated to calculate the bleb volume.

### Primary Outcome Measure

The primary outcome measure was the classification of bleb layer formation.

### Secondary Outcome Measures

The secondary outcome measures included relationships between the parameters of bleb images (bleb volume or classification based on the structure) and preoperative patient data, endplate position, and postoperative IOP.

### Statistical Analysis

Univariate comparisons between groups were performed using the Student *t*-test, Welch *t*-test, and the Mann–Whitney *U* nonparametric test. The correlation between bleb volume and postoperative IOP was analyzed using the Spearman rank correlation coefficient. *P* values < 0.05 were considered statistically significant. Multivariate analysis was performed to determine the prognostic factors for bleb structures using the Logistic regression model.

## Results

### Patient Characteristics

Sixty-two patients were enrolled in the present study. Two patients withdrew from the study because of poor physical condition at the time of MRI. In eight patients, the quality of MRI scans was determined as insufficient for the quantification of bleb areas and volumes because of body motion. Therefore, their data were excluded from the study. In total, 52 blebs were evaluated. The mean ± standard deviation length of time between surgery and MRI was 15.2 ± 10.1 months. Table 1 summarizes the baseline characteristics of patients and the position of the Baerveldt glaucoma implant. In 40 out of 52 eyes, the endplate was placed in the superotemporal quadrant.

**Table 1:** Patient characteristics

Characteristics	Total n = 52
Age, mean (SD), years	70.0 (10.5)
Sex, n (%)	
Men	24 (46)
Women	28 (54)
Type of glaucoma, n (%)	
Primary open-angle glaucoma	16 (31)
Exfoliation glaucoma	14 (27)
Neovascular glaucoma	9 (17)
Other secondary glaucoma	12 (23)
Primary angle-closure glaucoma	1 (2)
Preoperative IOP, mean (SD), mmHg	31.2 (9.8)
Number of preoperative glaucoma medications, mean (SD)	3.5 (1.0)
Postoperative IOP, mean (SD), mmHg	13.6 (4.1)
Number of postoperative glaucoma medications, mean (SD)	1.6 (1.8)
Axial length, mean (SD), mm	25.0 (2.9)
Number of previous intraocular surgeries, mean (SD)	2.4 (1.2)
Tube insertion position, n (%)	
Anterior chamber (BG101-350)	43 (83)
Pars plana (BG102-350)	9 (17)
Endplate position, n (%)	
Superotemporal	40 (77)
Inferotemporal	6 (11)
Superonasal	5 (10)
Inferonasal	1 (2)

IOP = intraocular pressure; SD = standard deviation.

### Primary Outcome Measure

**Figure 1:**
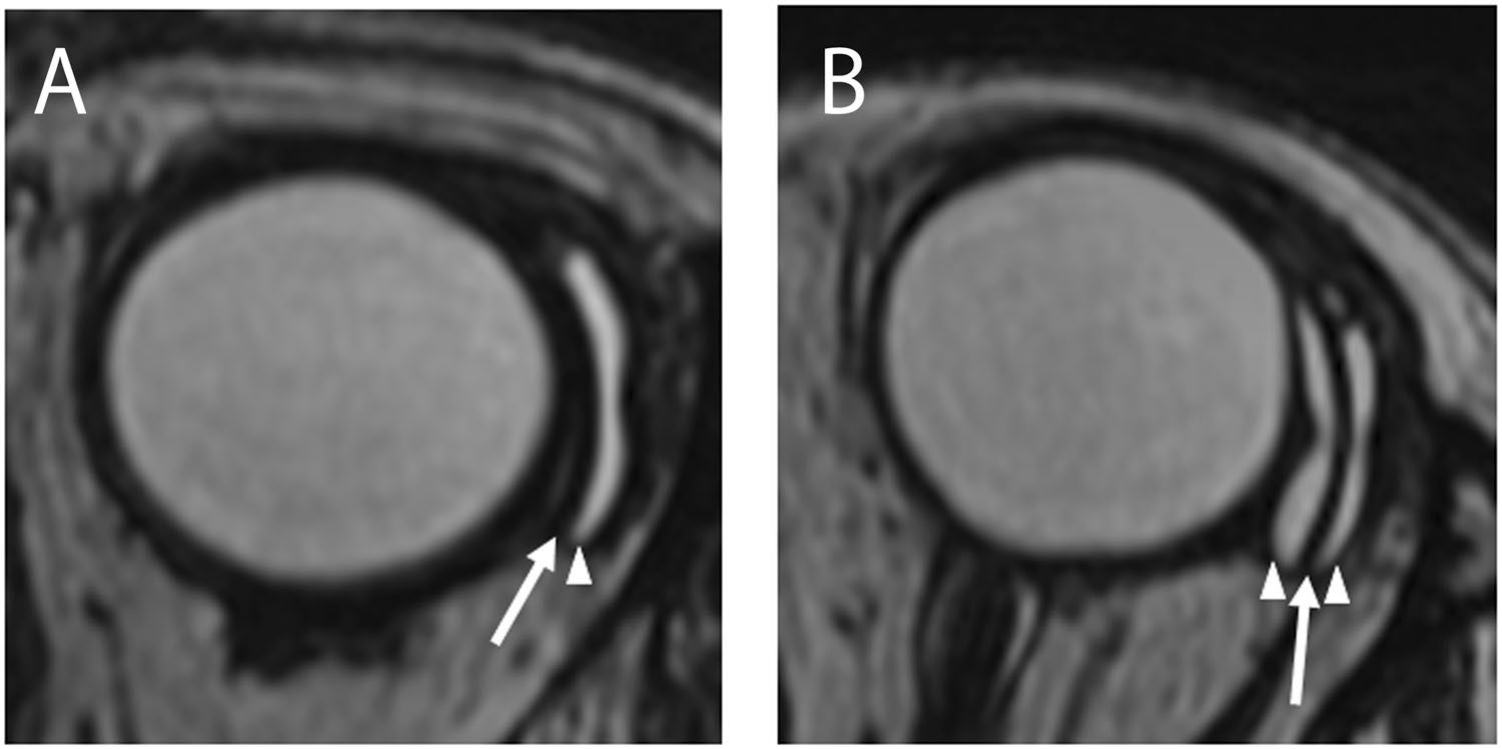
Magnetic resonance imaging scans of eyes with the Baerveldt glaucoma implant. (**A**) A case with a single bleb layer. (**B**) A case with a double bleb layer. A curvilinear low-intensity area was observed at the location of the endplate (arrows). A high-intensity area corresponding to the signal intensity of the aqueous humor, was located adjacent to the endplate (arrowheads).

**Figure 2:**
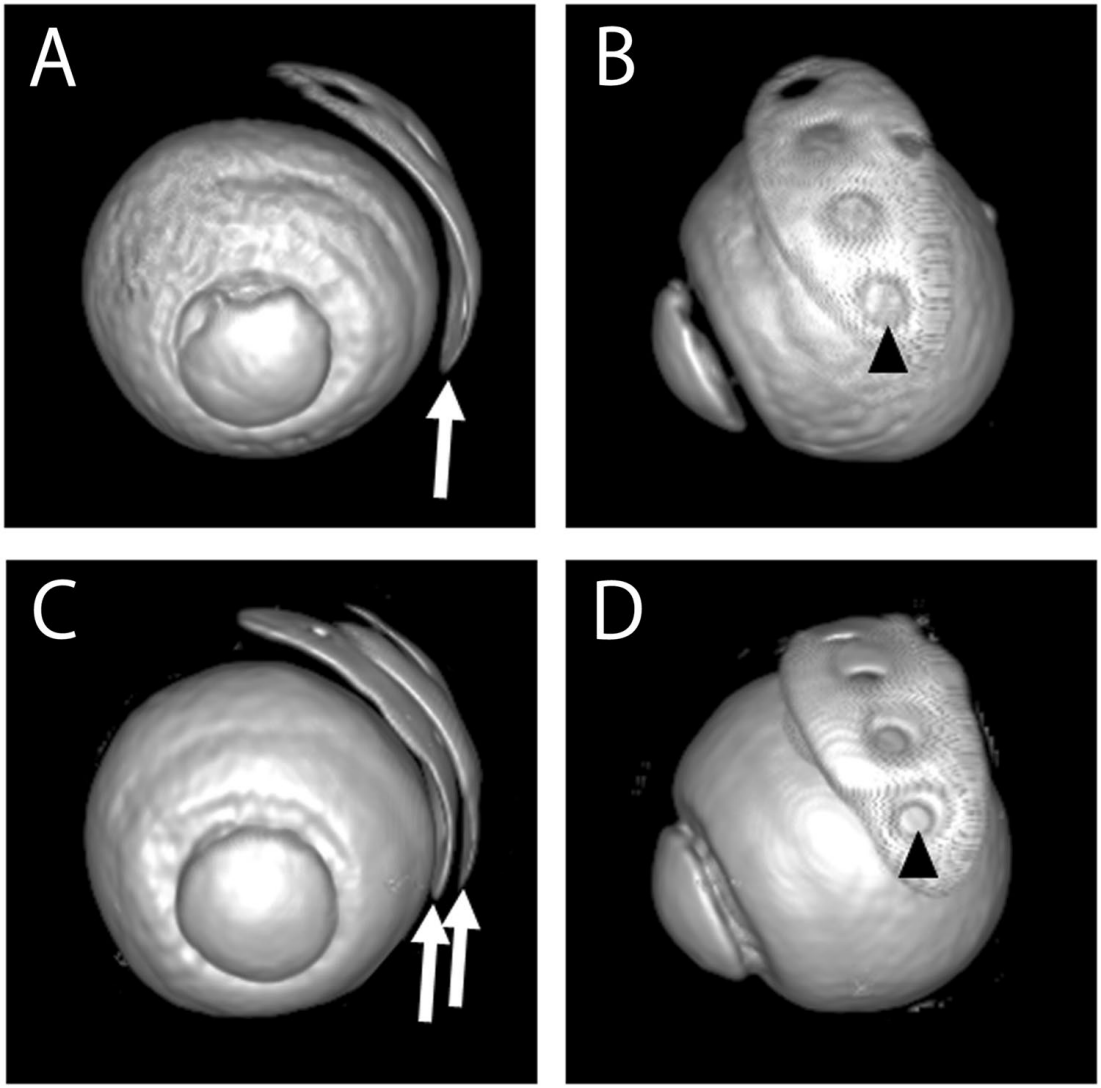
Three-dimensional magnetic resonance imaging scans of eyes with the Baerveldt glaucoma implant. Twenty-eight out of 52 eyes (53.8%) had bleb fluid on the outside of the endplate, and these eyes were classified into the single bleb layer group (**A**,**B**). The remaining 24 eyes (46.2%) had bleb fluid layers on each side of the endplate, and these eyes were classified into the double bleb layer group (**C,D**). Arrows indicate the bleb fluid layers. In both groups, four signal-free holes were observed in each bleb layer. Arrowheads indicate signal-free holes.

In all cases, a curvilinear low-intensity band that corresponded to the signal intensity of the silicone material was observed at the location of the endplate. Areas with the same high intensity as the intraocular fluid, corresponding to the signal intensity of the aqueous humor in the filtering bleb, were located adjacent to the endplate. In 28 out of 52 eyes (53.8%), the signal corresponding to the aqueous humor in the filtering bleb was detected on the outside of the endplate. The remainder of the eyes (46.2%) showed aqueous humor signals on both sides of the endplate. Based on this information, we classified the eyes into two groups: the single bleb layer group and the double bleb layer group (Fig. 1). Three-dimensional images were obtained in all cases. In both the single and double bleb layer groups, four signal-free holes were detected in each bleb layer. Because the endplate has four fenestrations, each signal-free hole was located on both sides of these fenestrations in the endplate (Fig. 2; Supplement Movie 1S).

### Three hundred sixty degree panoramas of magnetic resonance imaging scans of eyes with the Baerveldt glaucoma implant

(A) A 360-degree panorama of an eye in the single bleb layer group. (B) A 360-degree panorama of an eye in the double bleb layer group.

### Secondary Outcome Measures

#### Relationships between the parameters of bleb images and postoperative IOP

**Figure 3:**
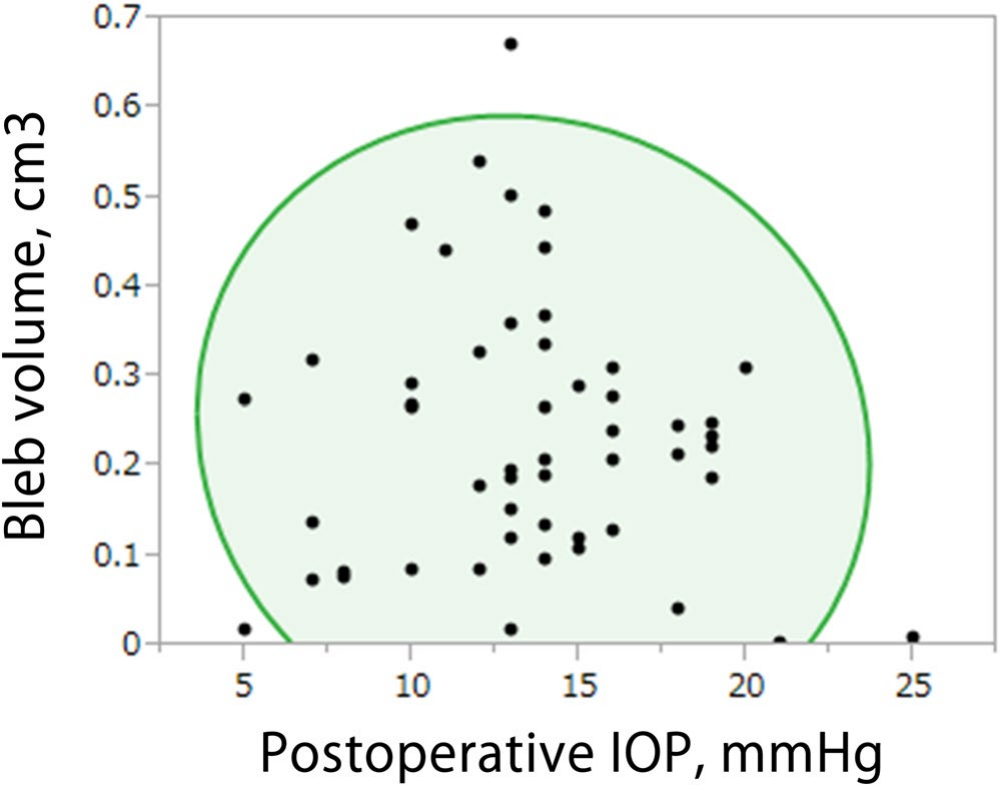
The correlation between postoperative intraocular pressure and bleb volume. The oval area shows a 95% bivariate ellipse. No significant correlation was found between postoperative IOP and bleb volume (Spearman rank correlation coefficient, *r* = −0.080; *P* = 0.57). IOP, intraocular pressure.

The mean ± standard deviation bleb volume was 0.23 ± 0.15 cm^3^ (n = 52). There was no significant correlation between bleb volume and postoperative IOP (*r* = –0.080; *P* = 0.57) (Fig. 3). There were no significant correlations between bleb volume and postoperative IOP in subgroup analyzes in both the single bleb layer group (*r* = 0.024; *P* = 0.90) and the double bleb layer group (*r* = 0.016; *P* = 0.94).

Comparisons of postoperative IOPs between the single bleb layer group and the double bleb layer group revealed significantly lower postoperative IOPs in the double bleb layer group than the single bleb layer group (12.3 ± 3.8 mmHg vs. 14.7 ± 4.1 mmHg, respectively; *P* = 0.033). The number of postoperative glaucoma medications taken did not differ significantly between the two groups (*P* = 0.87). Comparison of bleb volumes between the two groups showed a significantly larger mean bleb volume in the double bleb layer group than that of the single bleb layer group (0.28 ± 0.2 cm^3^ vs. 0.19 ± 0.1 cm^3^, respectively; *P* = 0.024).

#### Comparison of patient characteristics between the single bleb layer and double bleb layer groups

Table 2 shows patient data of the single bleb layer and double bleb layer groups. Among the preoperative data, there was a significant difference in the number of intraocular surgeries before Baerveldt glaucoma implantation between the two groups. The single bleb layer group had undergone more intraocular surgeries before Baerveldt glaucoma implantation than the double bleb layer group (2.8 ± 1.4 vs. 1.9 ± 0.8, respectively; *P* = 0.0068). However, there was no significant difference about details of prior surgeries between the groups (Supplement Table 1S). No significant differences were found in age, preoperative IOP, number of preoperative and postoperative glaucoma medications, axial length, length of time between surgery and MRI, type of glaucoma, tube insertion position, or the endplate position between the groups.

**Table 2:** Comparison of patient data between the single bleb layer and double bleb layer groups

	Single bleb layer (n = 28)	Double bleb layer (n = 24)	*P* value
Bleb volume, mean (SD), cm^3^	0.19 (0.1)	0.28 (0.2)	0.024
Age, mean (SD), years	69.1 (2.0)	71.0 (11.2)	0.53
Preoperative IOP, mean (SD), mmHg	30.9 (8.3)	31.4 (11.4)	0.79
Number of preoperative glaucoma medications, mean (SD), n	3.4 (1.1)	3.5 (0.8)	0.94
Postoperative IOP, mean (SD), mmHg	14.7 (4.1)	12.3 (3.8)	0.033
Number of postoperative glaucoma medications, mean (SD), n	1.5 (1.8)	1.6 (1.8)	0.87
Axial length, mean (SD), mm	25.2 (3.2)	24.9 (2.6)	0.80
Period between surgery and MRI, mean (SD), months	16.3 (11.1)	13.8 (8.9)	0.42
Number of previous intraocular surgeries, mean (SD), n	2.8 (1.4)	1.9 (0.8)	0.0068
Type of glaucoma, n (%)			1.00
Open-angle glaucoma	16 (57.1)	14 (58.3)	
Other glaucoma types	12 (42.9)	10 (41.7)	
Tube insertion position, n (%)			0.48
Anterior chamber	22 (78.6)	21 (87.5)	
Pars plana	6 (21.4)	3 (12.5)	
Endplate position, n (%)			1.00
Superotemporal	22 (78.6)	18 (75.0)	
Other	6 (21.4)	6 (25.0)	

IOP, intraocular pressure; MRI, magnetic resonance imaging; SD, standard deviation. Open-angle glaucoma includes primary open-angle glaucoma and exfoliation glaucoma.

### Determinants for the single/double bleb layer

Patient characteristics including age, preoperative IOP, number of preoperative and postoperative glaucoma medications, axial length, length of time between surgery and MRI, number of previous intraocular surgeries, type of glaucoma, tube insertion position, and endplate position, were evaluated as possible determinants for the formation of the single/double bleb layer. Multivariate analyzes using Logistic regression models showed that a higher number of previous intraocular surgeries was significantly associated with the formation of a single bleb layer (relative risk [RR], 2.85; *P* = 0.0014; Table 3). Conversely, the other factors were not significantly associated with the formation of a single/double bleb layer.

**Table 3:** Multivariate analysis for determining prognostic factors for the single bleb layer using the Logistic regression model

Variable	RR (95% Cl)	*P* value
Number of previous intraocular surgeries per each	2.85 (1.46–6.67)	0.0014
Preoperative IOP per mmHg	0.96 (0.87–1.03)	0.26
Age per year	0.99 (0.92–1.07)	0.80
Type of glaucoma (open-angle glaucoma/other)	1.23 (0.22–7.07)	0.81
Axial length per mm	0.85 (0.62–1.14)	0.27
Preoperative glaucoma medication per each	0.77 (0.36–1.55)	0.47
Postoperative glaucoma medication per each	0.90 (0.57–1.42)	0.65
Period between surgery and MRI per month	1.05 (0.97–1.13)	0.24
Tube insertion position (anterior chamber/pars plana)	3.39 (0.31–47.2)	0.32
Endplate position (superotemporal/other)	1.01 (0.21–4.88)	0.99

IOP, intraocular pressure; MRI, magnetic resonance imaging; RR, relative risk. Open-angle glaucoma includes primary open-angle glaucoma and exfoliation glaucoma.

## Discussion

The primary outcome of the present study was the characteristics of the bleb fluid structure in eyes that had undergone tube-shunt surgery using the 350-mm^2^ endplate Baerveldt glaucoma implant. The bleb fluid structure was characterized into two classes based on T2-weighted MRI scans: a single bleb fluid layer adjacent to the outer surface of the endplate (single bleb layer group) and a bleb fluid layer on either side of the endplate (double bleb layer group). Three-dimensional images demonstrated that each bleb fluid structure has four holes, which correspond to the four fenestrations of the endplate. While no significant correlation was found between the bleb volume and postoperative IOP (*r* = −0.080; *P* = 0.57), the double bleb layer group had a significantly lower mean postoperative IOP than the single bleb layer group (12.3 ± 3.8 mmHg vs. 14.7 ± 4.1 mmHg, respectively; *P* = 0.033). A higher number of prior intraocular surgeries before the Baerveldt glaucoma implantation was significantly associated with the formation of a single bleb layer (RR = 2.85; *P* = 0.0014).

Three previous studies had characterized and quantified bleb structure after tube-shunt surgery. Detorakis *et al*.^7^ analyzed bleb images of four eyes with the FP-7 Ahmed glaucoma valve and four eyes with the single-plate Molteno implant. Sano *et al*.^8^ analyzed bleb images of eight eyes with the FP-7 Ahmed glaucoma valve, 16 eyes with the 350-mm^2^ endplate Baerveldt glaucoma implant, and three eyes with the 250-mm^2^ endplate Baerveldt glaucoma implant. Additionally, Ferreira *et al*.^15^ analyzed 11 eyes with the FP-7 Ahmed glaucoma valve using MRI less than 6 months after surgery; however, it is important to note that postoperative IOP fluctuates within the 6 months after tube-shunt surgery^17^. Our present study is unique because the bleb images were evaluated in 52 eyes that had undergone tube-shunt surgery using the 350-mm^2^ endplate Baerveldt glaucoma implant more than 6 months after surgery. Moreover, we obtained bleb fluid images with thinner slices (0.6 mm) using a 3 Tesla scanner in combination with FIESTA-C. Our MRI conditions offer more detailed bleb images than the images from previous reports.

MRI data from the present study allowed distinct classification of the eyes into the single bleb layer and double bleb layer groups. Previous reports have indicated the existence of single and double bleb layers. In MRI scans from a previous study of eyes with the Ahmed glaucoma valve or the Baerveldt glaucoma valve, all eyes had the double bleb layer^8^. However, in another study, in MRI scans of 11 eyes with the Ahmed glaucoma valve, five eyes had bleb fluid between the sclera and the endplate as well as bleb fluid adjacent to the outside of the endplate^15^. The results of the present study suggest that the bleb structure in eyes with the Baerveldt glaucoma implant is similar to that in eyes with the Ahmed glaucoma valve. In the present study, the bleb fluid in all eyes with a single bleb layer was located adjacent to the outside of the endplate. Aqueous humor exits through the silicone tube from the tube opening on the outside of the endplate. The implant design may explain why bleb formation was more frequently found outside rather than inside the endplate.

Each bleb fluid has four holes, which correspond to the four fenestrations in the endplate. The old type of endplate did not have fenestrations^18^. The fenestrations are designed to allow the growth of fibrous columns through the bleb fluid. These fibrous columns connect the sclera and the bleb roof through the plate. It is believed that this design prevents the formation of oversized blebs, which could result in diplopia caused by ocular motility disturbance^4^. In fact, in a recent study using the Baerveldt glaucoma implant^19^, the frequency of diplopia seemed to be markedly lower than that in a previous report^20^. The results of the present study suggest that fenestrations contribute to the formation of fibrous columns.

We did not find a significantly negative correlation between postoperative IOP and bleb volume (*r* = −0.080; *P* = 0.57). Two previous studies showed significantly negative correlations between postoperative IOP and bleb volume^7, 8^. In contrast, another study did not find a negative correlation between postoperative IOP and bleb volume in eyes with the Ahmed glaucoma valve; in fact, they found positive correlations between postoperative IOP and the bleb height and length^15^. Two previous studies included more than two types of endplates, and both studies demonstrated a stronger IOP-lowering effect of the Baerveldt glaucoma implant than that of the Ahmed glaucoma valve^10, 11^. The IOP-lowering effect of the double-plate Molteno implant, which is more effective than the single-plate Molteno implant^9^, is comparable to that of the 350-mm^2^ Baerveldt glaucoma valve^21^. Because the endplate sizes of the Ahmed glaucoma implant and the single-plate Molteno implant are smaller than that of the 350-mm^2^ Baerveldt glaucoma valve, endplate size may be a confounding factor that is involved in the significantly negative correlation between postoperative IOP and bleb volume.

Eyes with double bleb layers exhibit lower IOPs than eyes with single bleb layers. At present, we cannot determine the reason why double bleb layers contribute to lower IOPs. A possible explanation is that the space between the endplate and the sclera in the eyes with the single bleb layer may be filled with fibrous tissue. Intense fibrosis causes bleb failure in filtering surgery^22–24^. The relatively high IOPs in the eyes with single bleb layers may reflect intense postoperative fibrosis in the filtering bleb. In fact, we found that a higher number of prior intraocular surgeries is a prognostic factor for the single bleb layer. Intraoperative conjunctival incisions cause fibroblast activation in the subconjunctival tissue^25^, and repeated ocular surgery may cause active fibrosis in the subconjunctiva, resulting in high postoperative IOP because of the single bleb layer.

The present study has some limitations, which are mostly related to the cross-sectional nature of the study. First, the endplate position was not standardized. Twelve out of 52 eyes had the endplate in the superonasal, inferotemporal, or inferonasal quadrants. It is possible that the choice of the endplate position depended on conjunctival scarring caused by prior surgical incisions. Having the endplate in quadrants other than the superotemporal quadrant appears to be associated with severe conjunctival scarring. However, our multivariate analyzes showed that having the endplate positioned in the superotemporal quadrant is not an independent factor for the formation of the double bleb layer. Second, we cannot discern whether the double bleb layer changes to a single bleb layer postoperatively, whether the double bleb layer is formed from the single bleb layer postoperatively, or whether the two types of bleb structures do not change during the postoperative follow-up period. A longitudinal study for each patient is required to clarify this limitation.

In conclusion, Baerveldt glaucoma implantation results in either a layer of bleb fluid on each side of the endplate or one layer outside the endplate. Each bleb fluid structure has four holes, which correspond to the fenestrations of the endplate. Lower postoperative IOPs are associated with the formation of the double bleb layer, and the larger bleb is likely associated with the lower IOP seen in eyes with the double bleb layer. Repeated intraocular surgery may cause the formation of the double bleb layer to fail, resulting in a higher IOP after Baerveldt glaucoma implantation.

## Electronic supplementary material

Supplement Table 1S

Supplement Movie A

Supplemental Movie B
