# Supplementary material for: Evaluation of Bleb Fluid After Baerveldt Glaucoma Implantation Using Magnetic Resonance Imaging
Source: Sci Rep. 2017 Sep 12;7:11345. doi: 10.1038/s41598-017-11054-x (PMC5595874; doi:10.1038/s41598-017-11054-x)
Supplement: Supplementary file 1 — Supplement Table 1S [file 41598_2017_11054_MOESM1_ESM.doc]

**Evaluation of Bleb Fluid After Baerveldt Glaucoma Implantation Using Magnetic Resonance Imaging**

Kentaro Iwasaki,^1^ Masayuki Kanamoto,^2^ Yuji Takihara,^1^ Shogo Arimura,^1^ Yoshihiro Takamura,^1^ Hirohiko Kimura,^3^ Masaru Inatani^1*^

**Author Affiliations**

^1^Department of Ophthalmology, Faculty of Medical Sciences, University of Fukui, Fukui, Japan

^2^Radiological Center, University of Fukui Hospital, Fukui, Japan

^3^Department of Radiology, Faculty of Medical Sciences, University of Fukui, Fukui, Japan

*** Corresponding author**

[AUTHOR]

Masaru Inatani

[POSTAL_ADDRESS]

23-3 Shimoaizuki, Matsuoka, Eiheiji, Yoshida, Fukui 910-1193, JAPAN

Phone No: +81-776-61-8400

Fax No: +81-776-61-8131

Email Address: [inatani@u-fukui.ac.jp](mailto:inatani@u-fukui.ac.jp)

Supplement table 1S. Comparison of previous intraocular surgeries data between the single bleb layer and double bleb layer groups

|  | Single bleb layer (n = 28) | Double bleb layer (n = 24) | *P* value |
| --- | --- | --- | --- |
| Number of previous intraocular surgeries, mean (SD), n | 2.8 (1.4) | 1.9 (0.8) | 0.0068 |
| Number of previous trabeculectomy, mean (SD), n | 1.1 (1.1) | 0.6 (0.5) | 0.20 |
| Number of previous trabeculotomy, mean (SD), n | 0.1 (0.3) | 0.1 (0.4) | 0.83 |
| Number of previous vitrectomy, mean (SD), n | 0.7 (1.3) | 0.3 (0.5) | 0.43 |
| Number of previous lens extraction mean (SD), n | 0.9 (0.3) | 0.8 (0.4) | 0.081 |
| Number of previous segmental buckling, mean (SD), n | 0.0 (0.0) | 0.08 (0.3) | 0.13 |

SD, standard deviation.
